# Spectroscopic characterization of rare events in colloidal particle stochastic thermodynamics

**DOI:** 10.3389/fchem.2022.879524

**Published:** 2022-08-12

**Authors:** Sandro K. Otani, Thalyta T. Martins, Sérgio R. Muniz, Paulo C. de Sousa Filho, Fernando A. Sigoli, René A. Nome

**Affiliations:** ^1^ Institute of Chemistry, State University of Campinas, Campinas, Brazil; ^2^ São Carlos Institute of Physics, University of São Paulo, São Carlos, Brazil

**Keywords:** stochastic thermodynamics, spectroscopy, rare events, Langevin, lanthanides

## Abstract

Given the remarkable developments in synthetic control over chemical and physical properties of colloidal particles, it is interesting to see how stochastic thermodynamics studies may be performed with new, surrogate, or hybrid model systems. In the present work, we apply stochastic dynamics and nonlinear optical light-matter interaction simulations to study nonequilibrium trajectories of individual Yb (III):Er (III) colloidal particles driven by two-dimensional dynamic optical traps. In addition, we characterize the role of fluctuations at the single-particle level by analyzing position trajectories and time-dependent upconversion emission intensities. By integrating these two complementary perspectives, we show how the methods developed here can be used to characterize rare events.

## Introduction

The application of fundamental concepts of nonequilibrium statistical mechanics to a variety of small systems (colloidal particles, RNA, DNA, or proteins, among others) has improved our understanding of energy conversion in the microscopic regime (Liphardt et al., 2002; Ciliberto, 2017). By studying fluctuations and probability distributions in out-of-equilibrium systems, stochastic thermodynamics has found applications in microscopic devices, biological systems and chemical reactions. For example, work fluctuations in nonequilibrium single-molecule measurements can be analyzed with fluctuation relations to extract important equilibrium information, such as the underlying free-energy landscapes of protein folding and DNA hairpin formation, among other biochemical processes (Nome et al., 2007; Arbore et al., 2019; Gieseler et al., 2021). Measurements of fluctuating thermodynamic quantities in small systems driven by temperature differences have been applied to study aging in glasses (Cugliandolo, 2011). Molecular motors driven by chemical reactions have been studied to characterize the efficiency and power production in such machines (Pietzonka et al., 2016).

Conversely, by treating these systems as the Brownian particle in a Langevin description of the underlying dynamics, experimental results have been used to verify theoretical predictions of stochastic thermodynamics, such as fluctuation theorems, the Jarzynski equality, and trajectory entropy (Seifert, 2012). Considering that nowadays it is possible to prepare colloidal particles with varying size, shape, chemical composition, and spectroscopic properties (Chen et al., 2014; Hueckel et al., 2021), it is interesting to explore such systems in connection with stochastic thermodynamics (Ciliberto, 2017; Bonança and Deffner, 2018; Saha et al., 2021). For example, large, thermal fluctuations can play an important role in individual stochastic realizations of a thermodynamic process, thus motivating the development of experimental methods for enhanced sampling of such rare events.

Previously, we have reported studies on stochastic dynamics and spectroscopy, including upconversion nanoparticles (UCNPs) (Nome et al., 2017a; Oliveira et al., 2019; Oliveira and Nome, 2019; Cavalcante et al., 2021; Oliveira et al., 2021). In reference (Nome et al., 2017a), we studied stochastic dynamics of co-doped Yb(III):Er(III) UCNPs. Upconversion spectra were used to characterize individual UCNPs, and nonlinear microscopy images based on wavelength-integrated upconversion luminescence were used to quantify stochastic trajectories of individual UCNPs in the presence and absence of optical trapping. The experimental results were compared with Langevin dynamics simulations in the presence of thermal, non-conservative, harmonic, and optical traps. In reference (Oliveira et al., 2021), we studied fundamental light-matter interaction mechanisms in core/triple shell UCNPs from experiments and simulations. Hierarchically structured Nd(III)-Yb(III)-Er(III) UCNPs were excited with CW and femtosecond laser-induced upconversion spectroscopy. The results were compared with light-matter interaction simulations for an 18-level system describing Nd(III)-Yb(III)-Er(III) photophysics over a time range spanning from femtoseconds to real-time.

The stochastic and spectroscopic approaches were combined in reference (Cavalcante et al., 2021) to study how nonlinear optical power laws in Yb(III)-Er(III) UCNPs may be characterized by individual stochastic trajectories in experiments and simulations. We studied UCNPs optically trapped, freely diffusing, and individual particles moving towards the static optical trap. In this way, we showed how stochastic dynamics might be helpful in characterizing UCNP photophysics at the single-particle level.

Here, we propose to study stochastic thermodynamics from a combined spectroscopic/colloidal particle perspective. We use the combined stochastic-spectroscopic approach as a starting point to develop methods for studying how the paradigmatic colloidal particle system of nonequilibrium stochastic thermodynamics is manifested in the nonlinear optical properties of the rare-earth doped colloidal particles. We characterize the role of fluctuations at the single-particle level by analyzing position trajectories and time-dependent upconversion emission intensities and comparing the information content obtained from stochastic and spectroscopic approaches.

## Methods

### Stochastic dynamics simulations

In the stochastic dynamics simulations, we solve the two-dimensional overdamped Langevin equation of motion for a Brownian particle, as described previously (Volpe and Volpe, 2013):

$$\frac{dx\left(t\right)}{dt}=\frac{1}{\gamma }F\left(x\right)+\sqrt{2D}W_{x}\left(t\right)$$ (1)

$$\langle W_{x}\left(t\right)W_{x}\left(t^{\prime }\right)\rangle =\delta \left(t-t^{\prime }\right)$$ (2)

with friction coefficient γ, diffusion coefficient D, delta-correlated white noise W_x_, Boltzmann constant k_B_, temperature T, and x = x,y (i.e., a symbol representing both spatial directions). The simulation parameters were chosen based on the experimental conditions described in reference (Cavalcante et al., 2021). Specifically, we use particle radius R = 1 μm, temperature T = 295 K, viscosity of water η = 10^-3^ Pa.s. Additionally for the dynamic trap simulations, the optical trap moves linearly in one dimension:

$$U\left(x,\lambda \left(t\right)\right)=\frac{1}{2}\kappa {\left(x-\upsilon t\right)}^{2}$$ (3)

λ(t)=υt (4)

In the two-dimensional optical trap, we studied trap stiffness in the range κ_x_ = κ_y_ = κ = 0.01 – 10 pN.μm^-1^, and pulling speed in the range υ = 0.5–100 μm.s^-1^. These simulation parameters were used to generate the results shown in Figures 2–4. For Figure 4, we also used particle radius R = 20 nm (Figure 3A) and trap stiffness κ_x_ = κ_y_ = κ = 0.2 pN.μm^-1^ (Figure 3B).

### Light-matter interaction simulations

**FIGURE 1:**
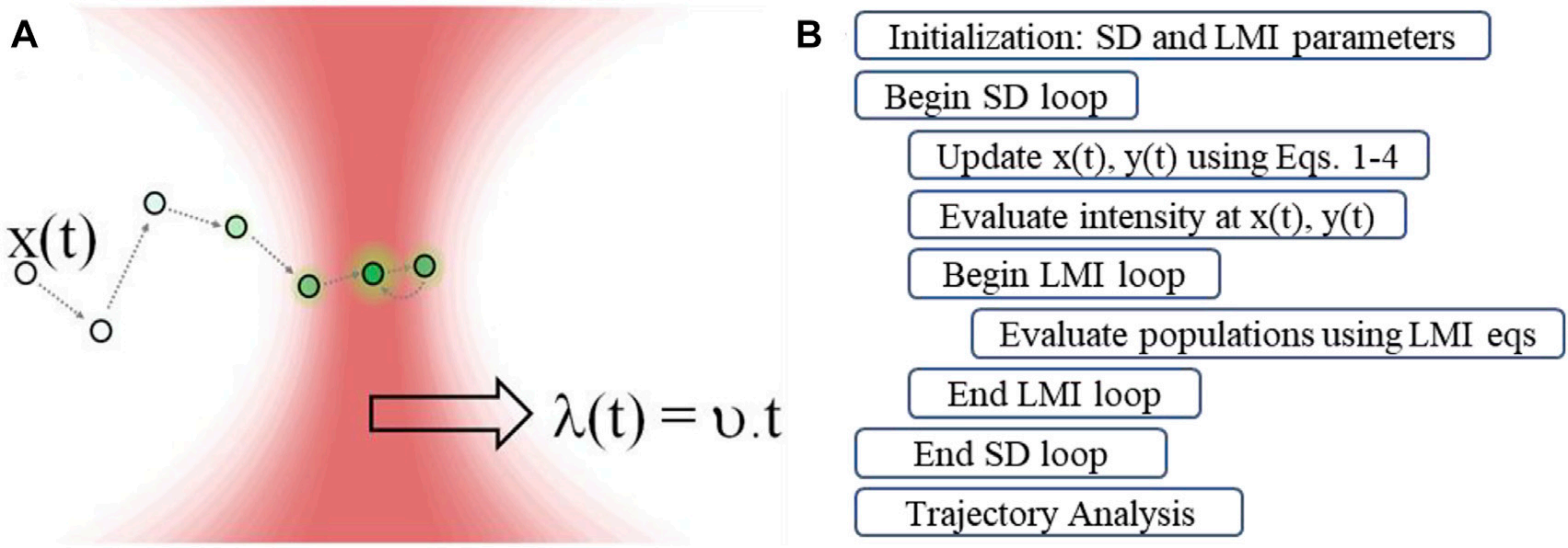
**(A)** Schematic description of the envisioned problem. **(B)** Outline of an algorithm for stochastic dynamics and light-matter interaction simulations.

**FIGURE 2:**
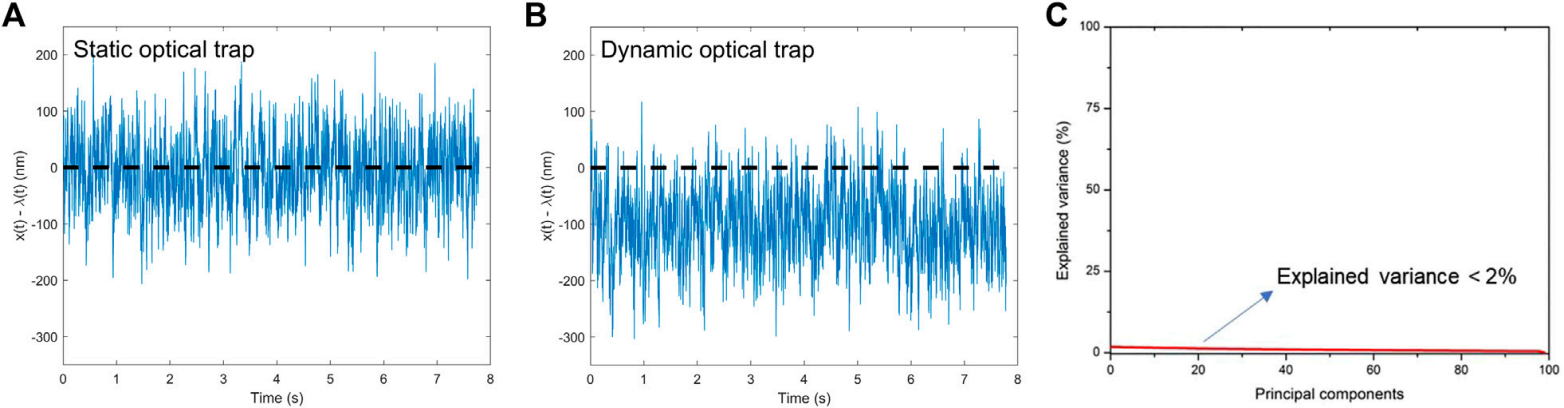
Stochastic trajectories for optically trapped colloidal particle. Simulation parameters: particle radius R = 1 μm, temperature T = 295 K, viscosity of water η = 10^−3^ Pa.s, trap stiffness κ_x_ = κ_y_ = 1 pN.μm^-1^. **(A)** Static optical trap; **(B)** Dynamic optical trap with pulling speed υ = 10 μm.s^-1^.

**FIGURE 3:**
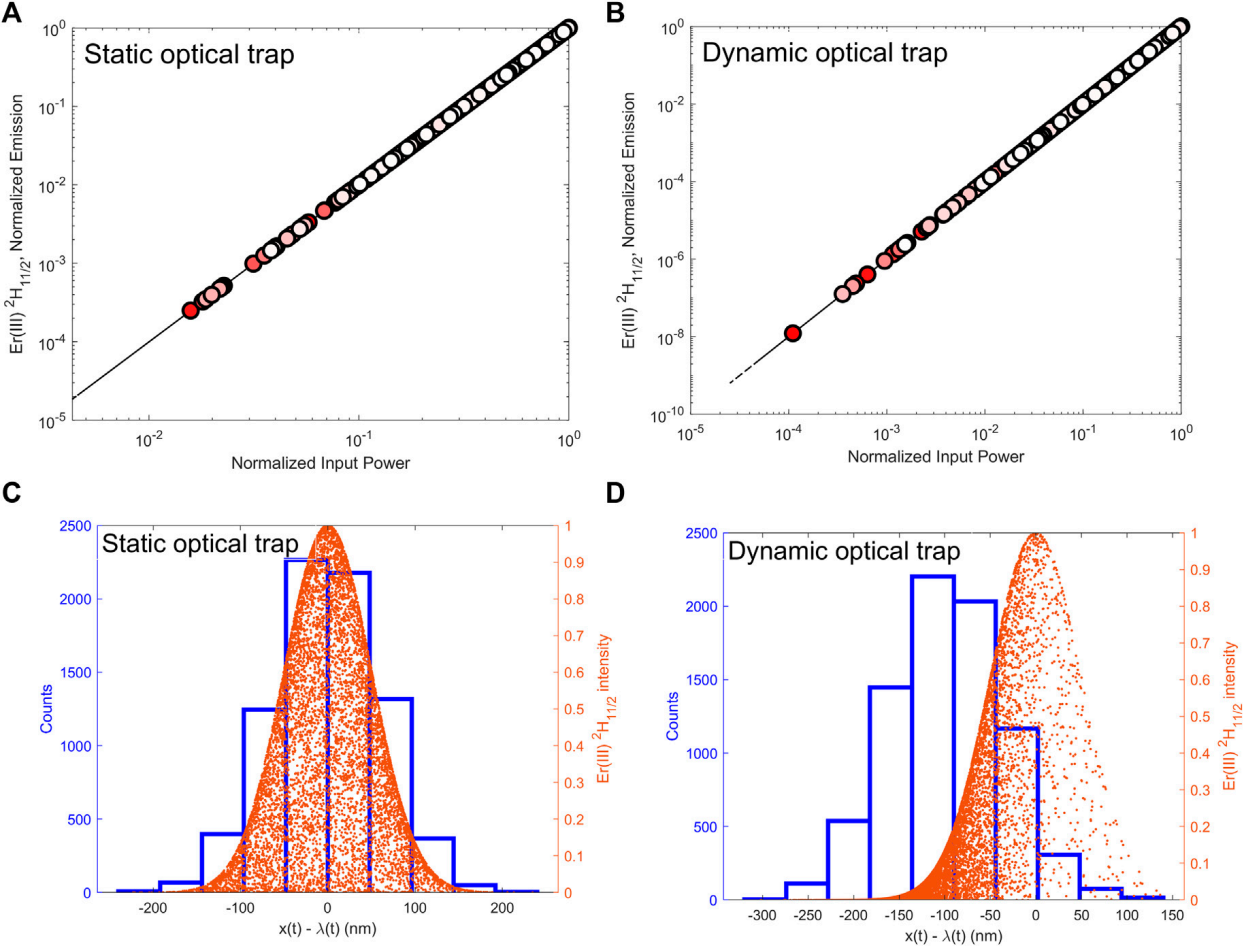
Top: On-the-fly calculation of nonlinear optical power law from individual stochastic trajectories: **(A)** Static optical trap; **(B)** Dynamic optical trap with pulling speed υ = 5.5 μm.s^-1^. The color gradient from red to white represents the simulation time (red corresponds to an early time, and white corresponds to a later time). Bottom: histogram of particle position (in blue) and the full set of emission intensities (orange dots) as a function of position: **(C)** Static optical trap. **(D)** Dynamic optical trap.

To describe the spectroscopic properties of the Brownian particle, we evaluate the rate expressions describing light-matter interactions for the Yb(III)-Er(III) 9 energy-level diagram and equations shown in the Supplementary Material (Oliveira et al., 2021). In this model, two levels describe Yb(III) and seven levels for Er(III) electronic states. First, we model light absorption by Yb(III) ^2^F_7/2_ state, non-radiative and energy transfer processes, followed by light emission, as described in refs (Yadav et al., 2013; Jung et al., 2015; Yadav et al., 2018; Diogenis et al., 2021; Oliveira et al., 2021). In the present work, we quantify upconversion emission from the population of the Er(III) ^2^H_11/2_. We initialized the starting ground state Yb(III) and Er(III) populations based on the experimental chemical composition of the UCNPs reported previously (Oliveira et al., 2021), and we used the remaining spectroscopic constants as described previously (see Oliveira et al., 2021). We used the Yb(III)-Er(III) 9 energy-level diagram shown in the Supplementary Material in the calculations related to Figures 1, 2, 3A,B. Additionally for Figures 3C,D, we used the corresponding energy level diagram for the pair Yb:Tm (Pires et al., 2004; Liang et al., 2019).

### Connecting stochastic trajectories with upconversion emission

For the coupled stochastic-spectroscopic dynamics simulations (Sule et al., 2015; Cavalcante et al., 2021), we begin by updating the particle position according to Eqs. 1–4. Next, we calculate the excitation light intensity at the updated particle location, assuming a two-dimensional Gaussian beam profile for the second, excitation laser, which is used as the excitation source for the spectroscopic transitions. Then, given the updated particle position and the excitation intensity at that updated position, we solve the light-matter equations for the Yb(III)-Er(III) energy-level diagram until the populations reach steady-state for each quantum state. Therefore, we used the steady-state populations calculated at each time step as an input for the next iteration in the stochastic dynamics loop. We use two loops to implement the integration of the stochastic dynamics and spectroscopic simulations. The outer loop performs the stochastic dynamics update and uses a time step dt_SD_ for a total simulation time t_end_. Here, dt_SD_ is a Brownian motion time step, corresponding to particle motion in the diffusive regime for the mass and friction parameters used in the present work. The inner loop performs the light-matter interaction update for each population, using a time step dt_LMI_ for a total duration dt_SD_, the time in which the energy level populations reach steady-state. At the end of the inner loop, we store the steady-state populations at the current simulation time and particle position. At the end of the outer loop, we store the stochastic trajectory Er(III) ^2^H_11/2_ population at each time step for further analysis. We calculated the Er(III) ^2^H_11/2_ population as a function of intensity on-the-fly as the Brownian particle moves in the presence of the time-dependent field described by Eqs. 3, 4. We studied the trajectories, the nonlinear optical power law, and histograms of particle position and upconversion emission. Figure 1B shows a flowchart of the coupled stochastic-spectroscopic method.

## Results and discussion

The main point of this work is to design an experiment to preferentially study rare events in nonequilibrium stochastic trajectories of an optically trapped upconverting Brownian particle. We study static and dynamic optical trapping of a single Brownian particle, a paradigmatic system in stochastic thermodynamics (Seifert, 2012). A single beam can be used for static optical trapping, whereas dynamic trapping may be achieved by controlling the flow or using beam steering techniques, such as acousto-optic deflection and spatial-light modulation. Additionally, we consider that the Brownian particle exhibits upconversion emission upon resonant excitation according to the energy level diagram for Yb(III)-Er(III) (see Methods section). Figure 1A shows the envisioned problem and Figure 1B shows a flowchart of the coupled stochastic-spectroscopic simulation method.

In this numerical study, we chose realistic parameters for the optical trap and the upconversion particle, as specified in the Methods section. We chose simulation parameters based on previously reported experimental work on optical trapping, upconversion nanoparticles, and stochastic thermodynamics. Thus, the method presented here builds upon previous work describing the combination of fluorescence-detected dual-beam optical tweezers to study structure and dynamics (Arslan et al., 2016; Comstock et al., 2016). We propose the use of dynamic optical tweezers and their application to observe upconversion nanoparticles under nonequilibrium conditions. First, we emphasize that optical trapping of upconversion nanoparticles has been described previously. For example, Haro-Gonzalez et al. (2013) reported using continuous-wave excitation at 980 nm to optically trap dielectric NaYF_4_:Yb(III)-Er(III) upconversion nanoparticles in distilled and heavy water (Haro-Gonzalez et al., 2013). Moreover, the combination of gradient forces, colloid chemistry and spectroscopy has been used to study fundamental aspects of optical trapping in upconversion nanoparticles. For example, Shan et al. (2021) studied resonance-enhanced, gradient-force optical trapping of low-refractive-index nanoparticles containing lanthanide ions at high doping concentration (Shan et al., 2021), and Ortiz-Rivero et al. (2020) reviewed synthesis/surface modification and optical trap volume reduction techniques used to increase optical forces in lanthanide-doped upconversion nanoparticles (Ortiz-Rivero et al., 2020). Although we focus on overdamped Brownian motion, we emphasize that ballistic dynamics of upconversion nanoparticles may be studied. For example, upconversion nanoparticles in solution in the presence of a temperature gradient and excited by a 980 nm laser moving in one dimension have been used to determine the Brownian particle velocity in the inertial regime (Brites et al., 2016). Recently, our group has used coupled stochastic-spectroscopic measurements and simulations to evaluate power laws for individual upconversion nanoparticle trajectories under the excitation intensity gradient of a static optical trap (Cavalcante et al., 2021). In the present work, we study how the power-law determined from this coupled approach can be used to improve the characterization of trajectories in stochastic thermodynamics, especially rare events.

Figures 2A,B show particle position trajectories calculated from Langevin dynamics simulations of optically trapped colloidal particles with static and dynamic optical traps, respectively. In our control setup (Figure 2A), we show the particle position trajectory as a function of time (blue line) when the particle undergoes Brownian motion in the presence of a static optical trap centered at zero (trap center indicated by the dashed black line). As shown in the Supplementary Material, the analysis of the trajectories shows that the mean and most probable particle position values are located at x = 0, as expected. On the other hand, Figure 2B shows x(t)–λ(t) (blue line), which is the difference between x(t), the particle position, and λ(t), the center position of the time-dependent harmonic trap for the protocol described by Eq. 4 with υ = 10 μm s^−1^ (see the Methods section). To facilitate visual comparison between Figures 2A,B, the position where x(t)–λ(t) = 0 is also shown with a dashed black line. The trajectory in Figure 2B thus shows that the average of the quantity x(t)–λ(t) assumes a non-zero value, unlike the trajectory calculated for the control system, as shown in Figure 2A. In other words, when x(t)–λ(t) < 0, as shown in Figure 2B, the particle lags the center of the time-dependent trap (Vaikuntanathan and Jarzynski, 2009). Nonetheless, the histogram associated with this trajectory also follows a Gaussian distribution—see Supporting Information (Seifert, 2012). Overall, Figures 2A,B illustrate how simulation parameters can be specified to achieve dynamic optical trapping of a lagging Brownian particle, such that rare events whereby the particle is located at the center of the time-dependent trap can be studied in a systematic way.

We have performed 100 independent simulations using the same system and setup parameters as in Figure 2B and longer simulation run, and the resulting trajectories are shown in the Supplementary Material. Visual inspection of this nonequilibrium ensemble already shows that all trajectories match the behavior observed in Figure 2B. In addition, in Figure 2C we used principal component analysis (PCA) to characterize the Gaussian nature of the relative position histogram distributions describing each trajectory (Souza and Poppi, 2012). The explained variance indicates the amount of information contained in each of the principal components, which we calculated in decreasing order of explained variance. For example, in the case of data sets that exhibit any type of tendency, the explained variance has high values, close to 100% for the first principal component. On the other hand, here we have obtained low values of explained variance for all principal components (Figure 2C). The results for explained variance by the principal components (Figure 2C) show that no tendencies are present in the data, since these values are extremely low (below 2%). In addition, the loadings analysis of the first three principal components (accumulated variance = 5.51%), presented in the Supplementary Material, clearly indicates that the information captured by these three principal components has no preferred variable. Therefore, although the optical trap moved linearly in one dimension with pulling speed υ, the calculated trajectories are described by Gaussian distribution. We thus conclude that the trajectories exhibit a Gaussian, uncorrelated stochastic behavior. As an additional consistency check, we have also calculated other stochastic thermodynamic quantities from the individual nonequilibrium trajectories: mean and standard deviation of work and dissipated heat, and the associated distributions. These quantities were also used to assess the role of negative work fluctuations on rare events.

In our coupled stochastic-spectroscopic simulation method, we explore two perspectives: using particle trajectories to study nonlinear optical response, and the other way around. In the first perspective, we studied how the spatially dependent excitation light intensity distribution can be used to probe the Brownian particle optical response. Specifically, starting from the particle position trajectories shown in Figure 2 and the corresponding excitation intensity at each particle position, we calculated the steady-state population of the Er(III) ^2^H_11/2_ state at each time step along the trajectory. From this set of excitation intensities and populations, we determined the single-particle nonlinear optical power law from individual trajectories. Figures 3A,B show the intensity-dependent upconversion emission power-law calculated on-the-fly from the position trajectories shown in Figures 2A,B, respectively. In Figures 3A,B, the results are shown with colored circles, where the color gradient from red to white represents the simulation time: red circle corresponds to early time (beginning of the simulation) and white circle corresponds to later time (end of the simulation).

In the double logarithmic plots shown in Figures 3A,B, the simulation results fall on a straight line, thus confirming the power-law equal to an exponent two, which is a nonlinear optical response expected for the system containing Yb(III)-Er(III) lanthanide ions. Therefore, in the stochastic trajectories calculated for static (Figure 3A) and dynamic (Figure 3B) optical traps, the Brownian particle is located in regions of lower and higher excitation light intensities. In comparing Figures 3A,B, we note that a broader intensity distribution is sampled in the dynamic trap (Figure 3B), due to the slope of the Gaussian excitation intensity profile at the center (Figure 3A) is small compared to the modulus of the slope one standard deviation away from the center (Figure 3B). Overall, Figures 3A,B are consistent with our previous work (Cavalcante et al., 2021), showing how particle trajectories can be used to study power laws. We emphasize that the simulation results shown in Figures 3A,B were obtained using a single input power, and the intensity gradient sampled by the diffusing particle enables the characterization of the power-law. Specifically, depending on the spatial location of the particle relative to the excitation beam spatial distribution, a lower or higher intensity is incident on the particle. On a timescale of the order of one Brownian time step (see Methods section), the population in each level of the Yb(III)-Er(III) system reached steady-state, including the state that we monitor, Er(III) ^2^H_11/2_. In this way, the emission intensities are dependent on the population of state Er(III) ^2^H_11/2_.

In Figures 3C,D we combine analysis of particle position histograms (relative to the trap center) and the corresponding upconversion emission intensities for the static and dynamic traps, respectively. In the case of the static trap, Figure 3C shows an overlap of the particle position histogram (blue) with the distribution of upconversion emission intensities (orange dots) of the upconverting particle. As expected, the particle spends more time at the center of the trap, which is also where the excitation and upconversion emission intensities are highest for the static trap. The overlapping distributions indicate that both approaches (particle position and upconversion emission) can be used to study particle trajectories in the case of the static optical trap.

Interestingly, for the dynamic trap, Figure 3D shows that the particle position histogram and the emission intensity distribution peak at different values of the quantity x(t)–λ(t). Specifically, the position histogram peaks at x(t)–λ(t) =−100 nm in Figure 3D, whereas the upconversion emission intensity distribution peaks at x(t)–λ(t) = 0. Therefore, while the particle spends most of the time in the location consistent with the dynamic harmonic trap pulling protocol, the upconversion emission intensity distribution follows the spatial distribution of the excitation light, such that the rare events in the histogram are excited where the source has the highest intensity. In this way, we show how to use the nonlinear optical response to study stochastic thermodynamics in a way that complements particle position-based analyses. Figure 3D is the main result of the present work and illustrates how the coupled stochastic-spectroscopic method can be used to characterize rare events in nonequilibrium stochastic trajectories with improved sensitivity and background-free detection by way of upconversion emission.

**FIGURE 4:**
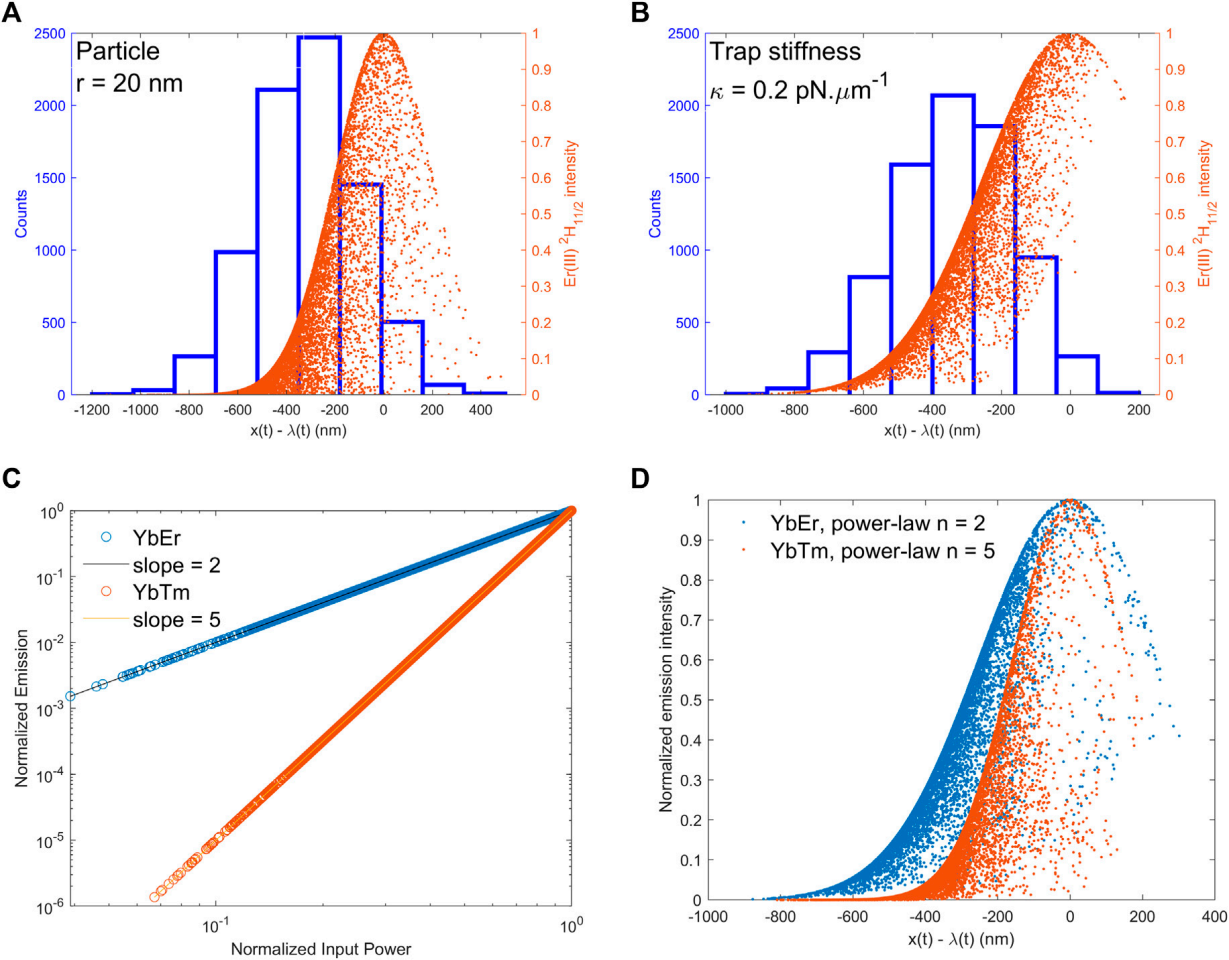
Top: Histogram of particle position (in blue) and full set of emission intensities (orange dots) as a function of position: **(A)** 20 nm radius nanoparticles in dynamic optical trap with υ = 5.5 μm.s^-1^; **(B)** 0.20 pN.μm^-1^ trap stiffness and υ = 5.5 μm.s^-1^. Bottom: Effect of chemical composition. **(C)** Nonlinear optical power law for YbEr (blue circles) and Tm (orange circles). **(D)** Full set of emission intensities for YbEr (blue dots) and Tm (orange dots) as a function of position.

Finally, we show that the proposed method for spectroscopic detection of rare events in single colloidal particle stochastic thermodynamics may also be realized with other values for the pulling protocol, excitation light, and particles of varying size and chemical composition. For example, Figure 4A shows application of the method using 20 nm radius particles (compare with R = 1 μm in Figures 2, 3), indicating emission distribution centered at x(t)–λ(t) = 0 and position histogram centered around x(t)–λ(t) = −300 nm. The effect of a smaller trap stiffness of 0.2 pN μm^−1^ (compare with 1 pN μm^−1^ in Figures 2, 3) is illustrated in Figure 4B, showing emission distribution centered at x(t)–λ(t) = 0 and position histogram centered around x(t)–λ(t) =−400 nm. Figures 4C,D compare two different models for upconversion emission based on YbEr and YbTm systems (Pires et al., 2004; Sigoli et al., 2006; Liang et al., 2019). Both models exhibit nonlinear optical responses that are well characterized with power-law equal to 2 for YbEr and 5 for YbTm, respectively, as shown in Figure 4C. Therefore, we further illustrate how the nonlinear optical response improves the sensitivity to rare event detection. The position histograms (Supplementary Material) for YbEr and YbTm overlap and are centered around the same value as before (see Figures 2, 3). On the other hand, the emission distributions are both centered at x(t)–λ(t) = 0, although with different shapes, thus clearly illustrating how the optical nonlinearity influences the observation of rare events.

## Conclusion

We have combined stochastic dynamics and nonlinear optical light-matter interaction simulations to study nonequilibrium trajectories of individual particles driven by two-dimensional dynamic optical traps. By using the nonlinear optical response of upconversion nanoparticles to improve the characterization of rare events, the method presented here has wide applications in stochastic thermodynamics. For example, the resulting trajectories can be used to study microscopic thermodynamics, including fluctuations in heat and work, trajectory entropy and the application of various fluctuation relations.

Starting from the integration of two complementary perspectives, we showed the advantage of using spectroscopic properties of a dynamically trapped Brownian particle to study nonequilibrium stochastic trajectories, particularly improving the characterization of rare events associated with negative work fluctuations. Furthermore, depending on the nature of the nonlinear optical response, an additional gain in rare event detection sensitivity can be achieved. The results presented here thus add to previous reports in which the opposite strategy was used, namely, the stochastic trajectories were used to improve the characterization of the nonlinear optical response of upconversion nanoparticles. In contrast, disadvantages of the present method include the increase in complexity of the system under study, compared with the canonical single colloidal particle paradigm of stochastic thermodynamics, and also increase in complexity of the simulations combining spectroscopy and Langevin dynamics.

Suggested improvements of this work include using a better model for the spectroscopic system, description of thermal effects, and considering the spatial averaging effect. The integrated stochastic-spectroscopic approach may also be extended to study other systems exhibiting nonlinear optical response, including dielectric micro-particles, plasmonic nanoparticles and nanorings (Nome et al., 2009; Nome et al., 2017b; Ferbonink et al., 2018).

## Data Availability

The original contributions presented in the study are included in the article/Supplementary Material, further inquiries can be directed to the corresponding author.
