# Supplementary material for: Spectroscopic characterization of rare events in colloidal particle stochastic thermodynamics
Source: Front Chem. 2022 Aug 12;10:879524. doi: 10.3389/fchem.2022.879524 (PMC9412910; doi:10.3389/fchem.2022.879524)
Supplement: Supplementary file 1 [file DataSheet1.docx]

Supplementary Material

Spectroscopic characterization of rare events in colloidal particle stochastic thermodynamics

Sandro K. Otani^1^, Thalyta T. Martins^2^, Sérgio R. Muniz^2^, Paulo C. de Sousa Filho^1^, Fernando A. Sigoli^1^, René A. Nome^1,*^

^1^ Institute of Chemistry, State University of Campinas, Campinas, Brazil

^2^ São Carlos Institute of Physics, University of São Paulo, São Carlos, Brazil

# * Correspondence: Corresponding Author nome@unicamp.br


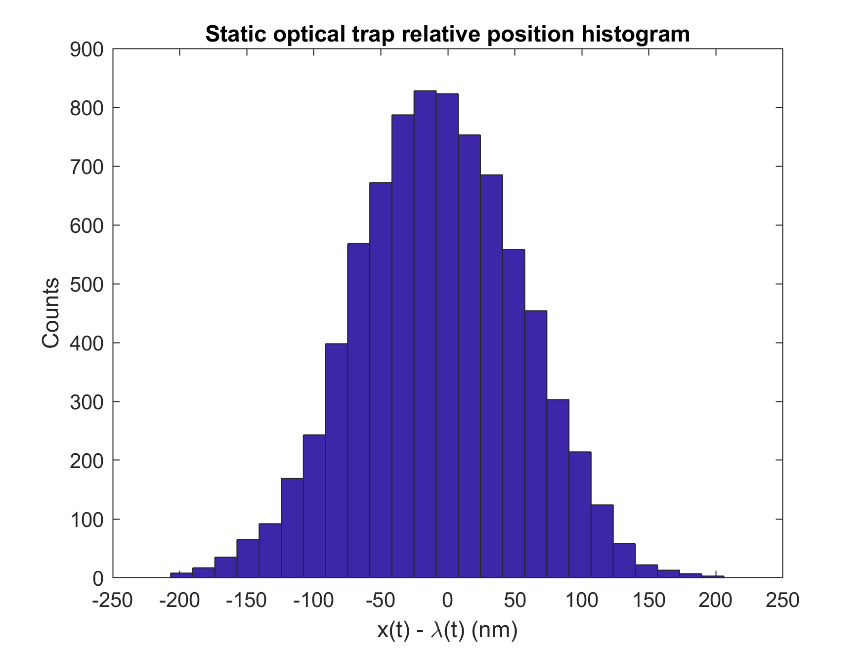


**Supplementary Figure 1.** Relative position histogram for the trajectory shown in Figure 2A of the main text.


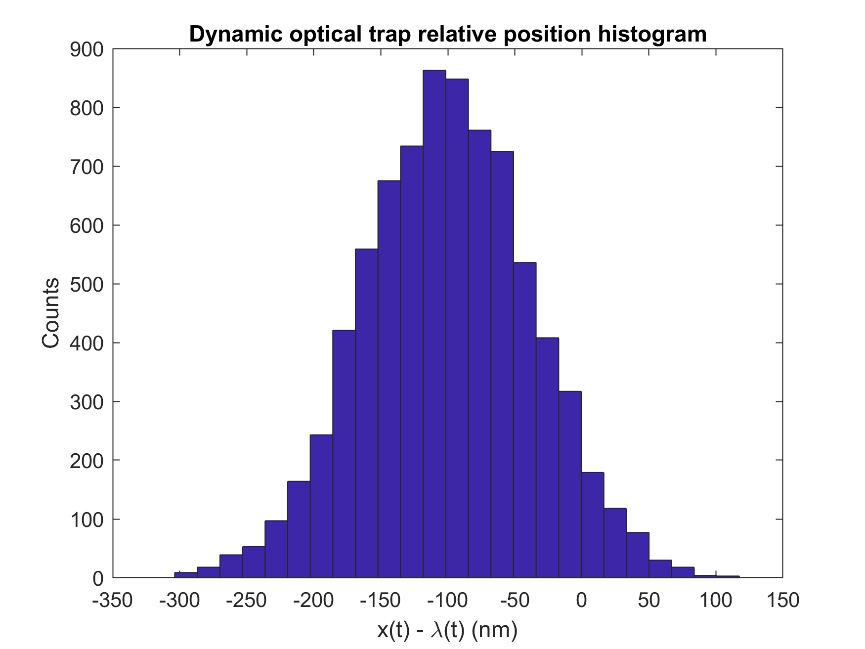


**Supplementary Figure 2.** Relative position histogram for the trajectory shown in Figure 2B of the main text.

Principal Component Analysis (PCA) is a Chemometric method widely used in exploratory data analysis^1^. The aim of this algorithm is to project data from a high-dimensional space to a reduced dimension, composed of new perpendicular axes, called Principal Components (PC), which describe the directions of greatest variance of the data^2^. Therefore, PCA identifies the presence of possible patterns or tendencies in the data by way of analysis of variance in the data set. Within the contexto of stochastic processes, the PCA method may be useful for further confirming the statistical distribution of the data set. Applied to the present work, we intended to confirm the Gaussian nature of the relative position histogram distributions for the calculated trajectories by showing that there are no tendencies in the trajectories as a function of time.

PCA consists of decomposing a matrix **X** containing *n* observations along the *m* variables, into a product of two matrices, Scores (**T**) and Loadings (**P**) plus a residuals matrix (**E**), Eq. 1

**X = T.P^t^ + E (Eq. S1)**

The Score matrix contains the observations of these samples in the PCA space and Loadings matrix contains the contribution of each original variable in this new projection.

| (A)  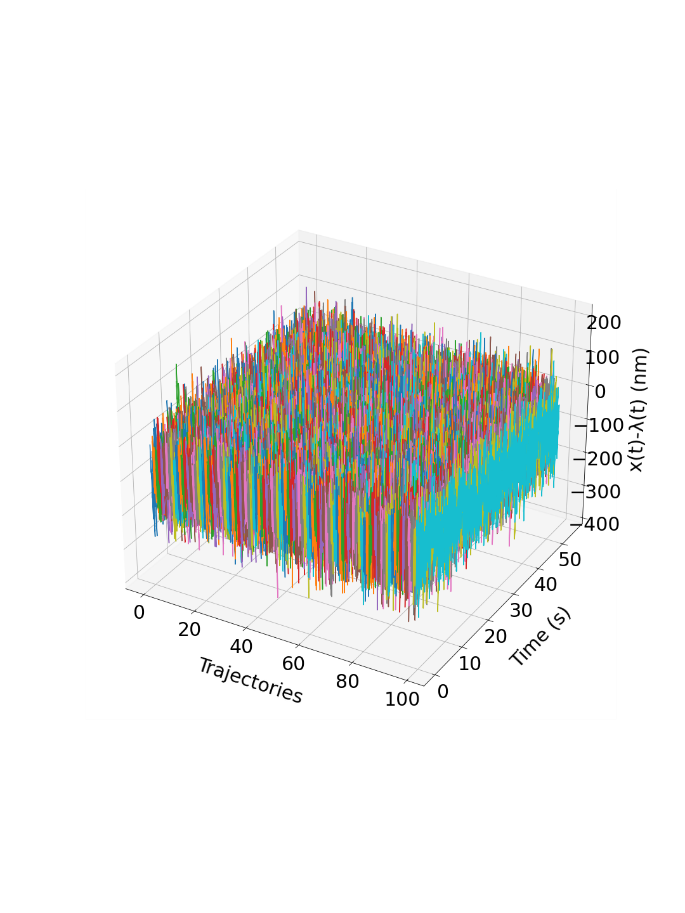 | (B)  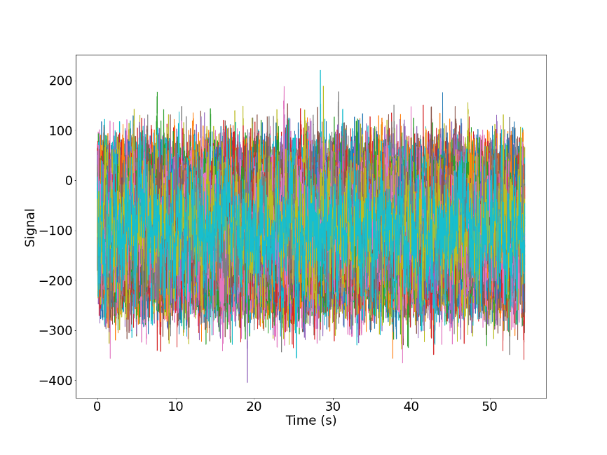 |
| --- | --- |

**Supplementary Figure 3.** Plot of 100 trajectories calculated with the same simulation parameter used in Figures 2A and 2B of the main text: (A) 3D view; (B) 2D.


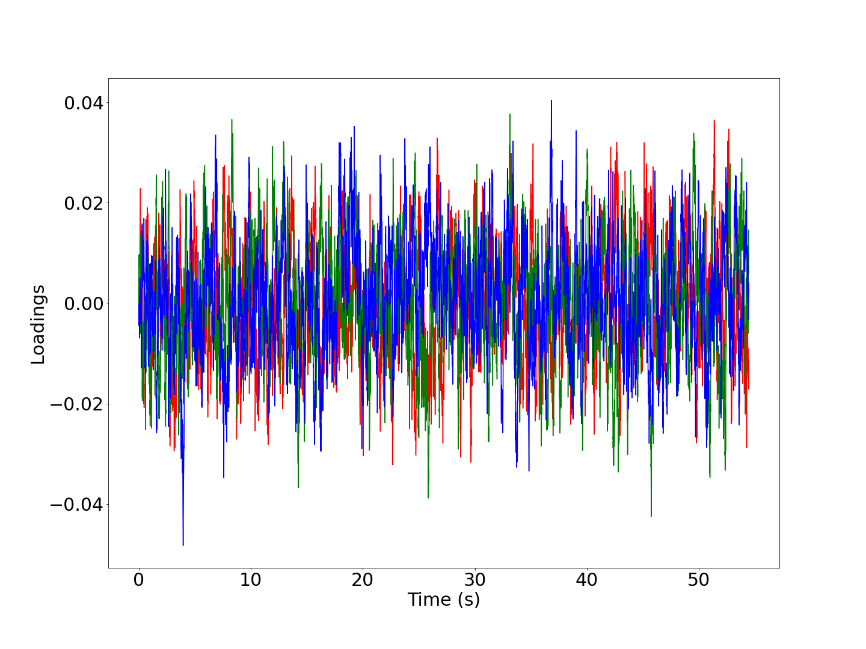


**Supplementary Figure 4.** Loadings of first three principal components.


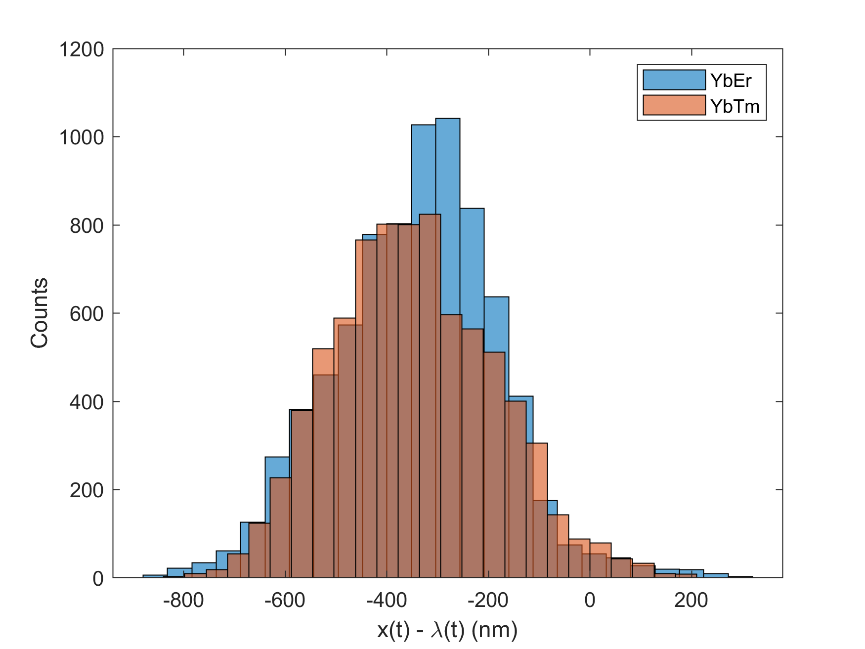


**Supplementary Figure 5.** Relative position histogram for the trajectories shown in Figure 4D of the main text for YbEr (blue) and YbTm (orange).


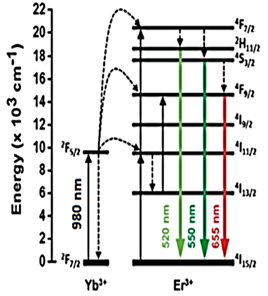


**Supplementary Figure 6.** Energy-level diagram for the pair of Yb^III^:Er^III^ ions studied in this work.
